# Neuroprotective effects of *Tradescantia spathacea* tea bioactives in Parkinson’s disease: *In vivo* proof-of-concept

**DOI:** 10.1016/j.jtcme.2024.01.003

**Published:** 2024-01-08

**Authors:** Lorenna E.S. Lopes, Sheilla da Silva Barroso, Joanny K.M. Caldas, Paulo R. Vasconcelos, Kirley M. Canuto, Claudio Dariva, Klebson S. Santos, Patricia Severino, Juliana C. Cardoso, Eliana B. Souto, Margarete Z. Gomes

**Affiliations:** a Tiradentes University (UNIT), Av. Murilo Dantas, 300, Aracaju, CEP 49032-490, Sergipe, Brazil; b Research and Technology Institute (ITP), Av. Murilo Dantas, 300, Aracaju, CEP 49032-490, Sergipe, Brazil; c Embrapa Agroindústria Tropical, Rua Dra. Sara Mesquita, 2.270, Bairro Planalto do Pici, Fortaleza, CEP 60511-110, Ceará, Brazil; d Laboratory of Pharmaceutical Technology, Faculty of Pharmacy, University of Porto, Porto, 4050-313, Portugal; e UCIBIO – Applied Molecular Biosciences Unit, MEDTECH, Department of Drug Sciences, Faculty of Pharmacy, University of Porto, Porto, 4050-313, Portugal; f Associate Laboratory i4HB - Institute for Health and Bioeconomy, Faculty of Pharmacy, University of Porto, Porto, 4050-313, Portugal

**Keywords:** Neuroprotective agents, Parkinson’s disease, Preclinical evaluation, Infusion, *Tradescantia*

## Abstract

**Background and aim:**

*Tradescantia spathacea* (*T. spathacea*) is a traditional medicinal plant from Central America and its tea, obtained by infusion, has been recognized as a functional food. The aim of this work was to investigate the effects of dry tea containing biocompounds from *T. spathacea* tea on motor and emotional behavior, as well as tyrosine hydroxylase (TH) and glial fibrillary acidic protein (GFAP) expression in 6-hydroxydopamine (6-OHDA)-lesioned rats.

**Experimental procedure:**

Bioactives were identified by Ultra Performance Liquid Chromatography (UPLC) and an *in vivo* study in male Wistar rats was run as proof of concept of neuroprotective effects of DTTS.

**Results and conclusion:**

We found 15 biocompounds that had not been previously reported in *T. spathacea*: the UPLC-QTOF-MS/MS allowed identification five phenolic acids, one coumarin, two flavonoids, one iridoid, one phenylpropanoid glycoside, and six fatty acid derivatives. The dry tea of *T. spathacea* (DTTS) presented significant antioxidant activity and high contents of phenolic compounds and flavonoids. Doses of 10, 30, and 100 mg/kg of DTTS were protective against dopaminergic neurodegeneration and exhibited modulatory action on the astrocyte-mediated neuroinflammatory response. Behavioral tests showed that 30 mg/kg of DTTS counteracted motor impairment, while 100 mg/kg produced an anxiolytic effect. The DTTS could be, therefore, a promising strategy for the management of Parkinson's disease.

## Highlights

• Dry tea of *T. spathacea* (DTTS) has high content of phenolic compounds and flavonoids.
• DTTS has significant antioxidant activity.
• DTTS is protective against dopaminergic neurodegeneration.
• DTTS exhibits modulatory action on the astrocyte-mediated neuroinflammatory response.
• Behavioral tests in rats show that 30 mg/kg of DTTS counteracted motor impairment.
• Behavioral tests in rats show that 100 mg/kg produced an anxiolytic effect.

## Graphical abstract

**Figure:**
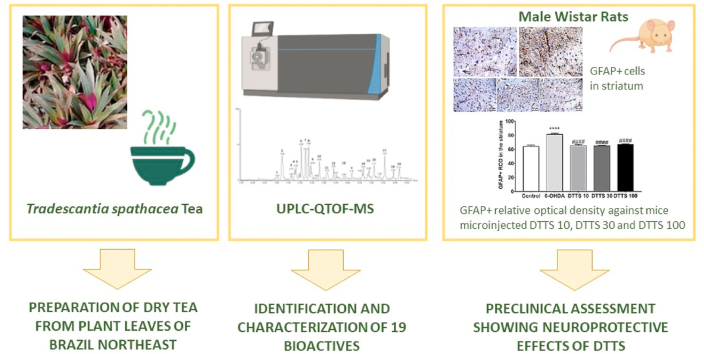

## Abbreviations

6-OHDA: 6-hydroxydopamine
ANOVA: One-way analysis of variance
BSA: Albumin bovine serum
CONCEA: Brazilian National Council for the Control of Animal Experimentation
DAB: 3,3-Diaminobenzidine
DPPH: 2,2-Diphenyl-1-picrylhydrazyl
DTTS: Dry tea of *T. spathacea*
ESI: Electrospray ionization
GABA: Gamma-aminobutyric acid
GFAP: Glial fibrillary acidic protein
MCP: Microchannel plate
PBS: Phosphate buffer saline
PD: Parkinson's disease
ROD: Relative optical density
TFC: Total flavonoid contents
TH: Tyrosine hydroxylase
TNF-α: Tumor necrosis factor-alpha
UPLC: Ultra Performance Liquid Chromatography

## 1 Introduction

Teas are functional drinks commonly used worldwide to treat or prevent diseases. These drinks have been object of study regarding the biological effects of their bioactive compounds.^1^ Teas can be obtained quickly by infusion, a conventional extraction method that uses hot water as a solvent and is associated with efficient extraction of polyphenols, compared to other techniques.^2^ The tea of *T. spathacea*, a herbaceous ornamental plant, is already popularly used as a functional drink in South and Central America and in Southeast Asia,^3^ and has the potential to be established internationally, due to its polyphenol content and antioxidant capacity.^4^

The aqueous extracts of *T. spathacea* show noteworthy antioxidant properties,^5^ similar to those of tocopherol and greater than ascorbic acid.^6^ Besides, it has been reported that *T. spathacea* extracts contain flavonoids in its phytochemical composition, such as kaempferol, quercetin, isoquercetin, luteolin-7-glucoside, rutin, epigallocatechin, and rhoeonin,^7^ as well as phenolic acids like vanillic acid, p-cumaric acid and ferulic acid.^5^

The flavonoids and phenolic acids from medicinal plants are bioactive compounds that have shown therapeutic potential in pathologies related to oxidative stress and inflammation,^8^ such as Parkinson's disease (PD). PD is a debilitating progressive illness characterized by the loss of dopaminergic neurons in the substantia nigra pars compacta, and consequent dopamine depletion in the striatum,^9^ and these biocompounds have shown neuroprotective actions in several studies using toxins to mimic PD physiopathology, such as the 6-hydroxydopamine (6-OHDA).^10^

Dopamine neuron pacemaker activity is crucial for maintaining dopamine levels; however, it can also lead to oxidative stress. Astrocytes protect neurons by antioxidant mechanisms, nevertheless, their function can be altered by microglia. Both microglia and astrocytes have dopamine receptors, which are more active during neuroinflammation in PD. Thus, studies have been focused on the antioxidative and anti-inflammatory effects of astrocytes and their synergism with microglia and dopamine.^11^^,^^12^

Polyphenols are important in PD due to an increase in the endogenous levels of antioxidant enzymes catalase, glutathione peroxidase,^13^ and superoxide dismutase.^14^ They also decreased lipid peroxidation^15^ and the tumor necrosis factor-alpha (TNF-α) production after the lesion of the nigrostriatal pathway,^16^ and presented neuroprotective effects against 6-OHDA-induced lesion *in vivo*^17^ and *in vitro*.^18^ Moreover, astroglial activation may impair dopaminergic neurons survival^19^ through the release of proinflammatory and neurotoxic factors, such as reactive nitric oxide, and some fatty acids and flavonoids with neuroprotective actions decreased glial fibrillary acidic protein (GFAP) immunoreactivity.^20^

Despite the biological properties of *T. spathacea* and its main compounds, the neuroprotective activity of *T. spathacea* has not been evaluated so far. Considering that abnormal protein accumulation and phosphorylation, mitochondrial dysfunction, deregulated kinase signaling, and greatly oxidative damage are key molecular mechanisms affecting the normal function and survival of dopaminergic neurons in PD,^21^ this study aimed to investigate the effects of the oral administration of dry tea obtained from the leaves of *T. spathacea* against behavioral and histological alterations induced by the monoaminergic toxin 6-OHDA in rats.

## 2 Materials and methods

### 2.1 Drugs and chemicals

6-hydroxydopamine (6-OHDA), ascorbic acid, apomorphine, 3,3-diaminobenzidine (DAB), primary antibodies, 2,2-diphenyl-1-picrylhydrazyl (DDPH) and hydrate rutin ≥94% were obtained from Sigma-Aldrich® (St. Louis, MO, USA), veterinary pentabiotic from Fort Dodge® (Campinas, Brazil), xylazine and ketamine from Syntec® (São Paulo, Brazil), hematoxylin from Merck® (Darmstadt, Germany), antibodies and peroxidase-labelled streptavidin LSAB2 kit from DAKO® (California, USA), gallic acid monohydrate PA from Neon® (Mumbai, India), Folin-Ciocalteau reagent from Dynamic® (Goiânia, Brazil).

### 2.2 Plant material

The *T. spathacea* leaves were collected from Aracaju, Sergipe, Northeast of Brazil (10°58′7.536″S, 37°3′28.469″W). It was registered an exsiccate at the Herbarium of the University Tiradentes-Aju (Protocol: n°. 0845). All leaves used in the extractions were dried at 45 °C for 96 h in a hot-air circulation oven. The leaves were then milled to the ranging size from 16 to 32 mesh using sieves of Tyler series. The humidity of samples was then measured (9.4 ± 0.4%), and the material was stored protected from light and room temperature in a refrigerator.

#### 2.2.1 Preparation of dry tea

The dry tea from *T. spathacea* (DTTS) was obtained according to previously reported^22^ with adaptations. Briefly, water at 100 °C (30 mL) was placed in *T. spathacea* leaves (1 g). After 15 min without further heating, the tea was filtered with filter paper and dried at 45 °C in an oven with hot-air circulation until obtaining a constant mass. The preparation of dry tea was performed in triplicate. The yield of the extraction process was estimated based on the percentage of dry mass obtained, which was calculated regarding the initial mass of *T. spathacea* before extraction.

#### 2.2.2 Determination of total polyphenol content

Total phenolics content (TPC) were determined by using the Folin-Ciocalteu method. Briefly, 0.5 mL of the sample was diluted to methanol (250 ppm) and mixed with 2.25 mL of Folin Ciocalteu solution, 1.75 mL of 7.5% sodium carbonate solution, and 0.5 mL of distilled water. After, the solution was incubated at 45 °C for 20 min. The absorbance of the mixture was measured at 765 nm using a UV–vis spectrophotometer (721 G visible spectrophotometer). The calibration curve was obtained from gallic acid standard (y = 0.0108x + 0.021), range from 5 to 140 μg/mL (R^2^ = 0.9997). Total phenolic values were expressed as mg of gallic acid equivalents by g of dry tea (mg GAE/g DT).

#### 2.2.3 Determination of total flavonoid content

The total flavonoid contents (TFC) in the dry tea were determined using the aluminum nitrate colorimetric method. Briefly, 250 ppm of the sample diluted to 0.5 mL of methanol was mixed with 0.1 mL of aluminum nitrate 10%, 0.1 mL of potassium acetate 1 M, and 4.3 mL of methanol. Then, the mixture was left to stand for 10 min at room temperature in the dark. Rutin was used as the standard for calibration curve (y = 0.0039x + 0.0033), range of 5–140 μg/mL (R^2^ = 0.9986). The absorbance at 425 nm (721 G visible spectrophotometer) was used for the determination of flavonoids content. The results were reported as mg of rutin equivalents per g of dry tea (mg RE/g DT).

#### 2.2.4 DPPH free radical scavenging assay

For the DPPH assay, DTTS (5 mg) was previously diluted in methanol (10 mL) to obtain a stock solution at a concentration of 500 μg/mL 300 μL of DTTS at distinct concentration (100, 50, 25, 12.5, 6.2 μg/mL) was mixed with 2700 μL of the DPPH solution (0.06 mM). The mixtures were homogenized and held for 10 min at ambient temperature without light incidence and the absorbance was measured at 517 nm (visible spectrophotometer of 721 G). The control was performed using only the DPPH solution. The results were expressed as the IC50, i.e., the amount of antioxidants needed to reduce the initial concentration of radicals by 50%. IC50 values were calculated using a nonlinear regression curve from Prism software 7.0 software.

#### 2.2.5 UPLC-ESI-QTOF-MS analysis

Ultra-Performance Liquid Chromatography (UPLC) analyses were performed on an Acquity UPLC (Waters, USA) system coupled to a Xevo QTOF mass spectrometer (Q-TOF, Waters). Separations were performed on a C18 column (Waters Acquity® UPLC C18; 150 mm × 2.1 mm, 1.7 μm). For metabolic fingerprinting, a 2 μL aliquot of the tea was subjected to UPLC analysis using an exploratory gradient with a mobile phase comprising deionized water (A) and acetonitrile (B), both containing formic acid (0.1% *v/v*). The tea was subjected to the exploratory gradient as follows: 2%–95% for 15 min, at a flow rate of 500 μL min^−1^. Ionization was performed with electrospray ionization (ESI) source in negative ion mode, in the range of 110–1200 Da. The optimized instrumental parameters were as follows: capillary voltage of −2800 V, cone voltage of −40 V, source temperature of 120 °C, desolvation temperature of 330 °C, flow cone gas of 20 L h^−1^, desolvation gas flow at 600 L h^−1^, and microchannel plate (MCP) detector voltage of −1900 V. The mode of acquisition was MS^E^, and the system was controlled using MassLynx 4.1 software (Waters Corporation).

### 2.3 Biological assay

#### 2.3.1 Animals

Forty adult male Wistar rats weighing between 180 and 250 g were housed under a 12 h/12 h light/dark cycle in a controlled temperature room (21 ± 2 °C), with food (rat chow, Labina® Purina, São Paulo, Brazil) and water *ad libitum*. All experiments were approved by the Animal Experimentation Ethics Committee of the Tiradentes University (protocol number 020517) and performed according to the Brazilian National Council for the Control of Animal Experimentation (CONCEA). The experiments were designed following the 3Rs policies.

#### 2.3.2 Experimental design

After surgical procedures for the intrastriatal injection of 6-OHDA, described above or saline, the animals were divided into groups (n = 8 per group), identified as: Control (microinjected with ascorbate saline and treated with distilled water), 6-OHDA (microinjected with 6-OHDA and treated with distilled water), DTTS 10, DTTS 30 and DTTS 100 (microinjected with 6-OHDA) and treated daily by oral gavage dosing of *T. spathacea* dry tea at 10, 30 or 100 mg/kg, respectively, for 30 days. The open field test, cylinder test, and the evaluation of rotational behavior tests were performed over time, as indicated below. Then, the animals were euthanized 30 days after surgery and their brains were dissected and processed for histological analysis (immunohistochemistry for tyrosine-hydroxylase and glial fibrillary acidic protein).

#### 2.3.3 Unilateral 6-OHDA lesions

Rats were deeply anesthetized with 1 mL of xylazine (100 mg/kg, i.p.) plus 1 mL of ketamine (10 mg/kg, i.p.s). They were fixed in a stereotaxic frame (Insight, Brazil) and received 3 μL (6.7 μg/μL) of 6-OHDA diluted in 0.02% ascorbic acid and distilled water, injected in the right striatum at a rate 1 μL/min with an infusion pump (Insight, Brazil), using a 10 μL Hamilton syringe. Animals of the control group received the same volume of ascorbate saline. The stereotaxic coordinates were: AP: +1.0 mm; LL: +3.0 mm; DV: −5.0 mm.^23^ The needle was kept in place for an additional 5 min to avoid reflux. After surgical procedures, the animals were placed under a heating lamp for 1 h and received veterinary pentabiotic (0.2 mL/kg, i.m.) to prevent infection.

#### 2.3.4 Open field test

The assessments were performed at 24 h and 15 days after surgery. The open field apparatus was made of white-colored wood, and it consisted of a quadrilateral with an area of 4830.25 cm^2^ and walls that were 34.5 cm high, with the base subdivided into sixteen quadrants visibly marked by black lines. The locomotion (crossing) and rearing (the number of times the rat stood on its hind legs) parameters, as well as the fecal bolus, were evaluated for 5 min.

#### 2.3.5 Cylinder test

Tests were carried out at 9 and 29 days after intrastriatal injections. The number of spontaneous ipsi or contralateral forelimb activity while rearing against the wall of the arena (open top, transparent plastic, 30 cm high and 6 cm diameter) was recorded for 5 min. Two mirrors were placed behind the apparatus, to keep a 360° vision. The number of impaired and non-impaired forelimb contacts was calculated as the percentage of total contacts.

#### 2.3.6 Apomorphine-induced rotation behavior

The test was performed 30 days after the beginning of the experiments. It was carried out in a circular arena with 31 cm of diameter and 13 cm high. After a subcutaneous, injection of 0.5 mg/kg apomorphine, the total number of ipsilateral rotations was counted for 60 min.

#### 2.3.7 Immunohistochemistry

Histological sections of 5 μm thick from paraffin-embedded and formalin-fixed samples were deparaffinized in xylene and dehydrated in increasing alcohol concentration solutions (90 and 100%). After washing with phosphate buffer saline (PBS), the antigen retrieval was performed using 0.1 M citrate buffer, pH 6.0, in a microwave on high power (three cycles, 5 min each). Endogenous peroxidase was blocked using 1% H_2_O_2_ solution in PBS for 7 min. To reduce nonspecific background staining, tissue sections were incubated with albumin bovine serum (BSA, 5% in PBS) for 60 min. Then, they were incubated overnight with the primary antibodies (tyrosine hydroxylase - TH antigen produced in mouse, monoclonal, clone TH-16, diluted at 1:500 in PBS 0.1 M, Triton X-100 and albumin bovine serum 5%; glial fibrillary acidic protein - GFAP antigen produced in mouse, clone S206A-8, diluted at 1:100 in PBS 0.1 M, Triton X-100), followed by 2 sequential 30 min incubations with biotinylated link antibody and peroxidase-labelled streptavidin, respectively, according to the instructions of the manufacturer (kit DAKO). Staining was completed after 10 min incubation with 10 mg of DAB diluted in 20 mL 0.3% H2O2 prepared in PBS. Counterstaining was performed using Mayer hematoxylin. Sections were then rehydrated, clarified in xylene, and covered with balm and coverslips.

#### 2.3.8 Image analysis

Immunostaining was considered positive when a brown color was visible in the soma, dendrites, and axons of neurons. Also, glial cells (astrocytes) positively stained presented a brown color reaction product in their cytoplasm. The equipment consisted of a BIOPTIKA B60 microscope, equipped with a 100X objective and connected to a photographic camera CMEX. The captured images were analyzed by the software Image J, from the public domain of the National Institute of Mental Health. The number of TH + cells was counted both *ipsi* and contralateral SNc.^24^ Labeling of TH+ and GFAP + fibers in the striatum were assessed by the relative optical density (ROD), by measuring average pixel optical density over cell body and network in grayscale from 0 to 255, being 0 the highest intensity and 255 the lower.^20^ The dorsolateral, dorsomedial, and ventrolateral regions of the striatum (both *ipsi* and contralateral to the lesion) were analyzed and a mean value was obtained. The ROD of the lateral ventricle in each section was considered as a background (control) and values were divided by this background. The counts and measurements were performed in 4 ± 1 section and then we calculate the mean value per rat. The results were expressed as a percentage of the contralateral side.

### 2.4 Statistical analysis

Data were expressed as mean ± SEM. Gaussian distributions of ordinal data were assessed by the Shapiro-Wilk test. Then, behavioral data from the open field and cylinder tests were analyzed by two-way analysis of variance with repeated measures (factors being treatment and time), and every other data (histological) were analyzed by one-way analysis of variance (ANOVA). The Bonferroni post-test was always applied to verify differences between groups. The Kruskal-Wallis with Dunn's post-test was applied to analyze the rotational behavior. Values of p < 0.05 were considered to be statistically significant. The degrees of freedom were designed as F. All statistical analyses were performed using GraphPad Prism (version 8.0).

## 3 Results

### 3.1 Chemical characterizations of DTTS

In the present study, dry tea of *T. spathacea* leaves was obtained by infusion with an overall yield rate of 21.5 ± 0.4%. Moreover, DTTS showed the amount of TPC of 31.7 ± 1.5 mg GAE/g, and TFC of 29.1 ± 0.5 mg RE/g. We found an antioxidant activity with an IC_50_ value of 16.7 ± 1.9 μg/mL.

**Fig. 1:**
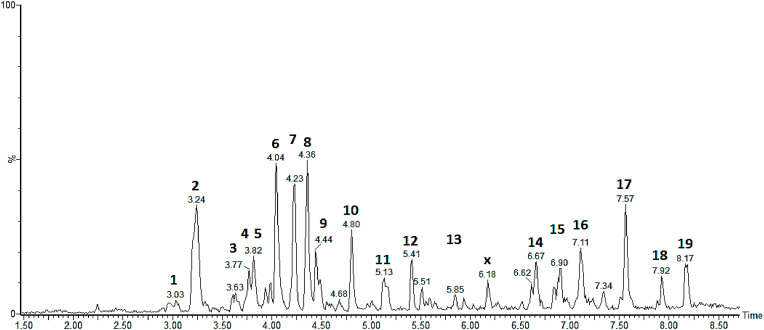
UPLC-QTOF-MS^E^ chromatogram, negative mode, in *Tradescantia spathacea* tea. Peaks are identified in Table 1.

In order to identify the biocompounds present in the dry tea, it was performed the UPLC-QTOF-MS/MS in negative-ion MS spectra (Fig. 1). The 19 compounds identified can be seen in Table 1.

**Table 1:** Identification of biocompounds in *Tradescantia spathacea* tea (UPLC-QTOF-MS/MS in negative-ion MS spectra).

Peak no.	Rt min	[M − H]^-^ Observed	[M − H]^-^ Calculated	Product Ions (MS/MS)	Empirical Formula	Ppm (error)	Compound	Reference
**1**	2.98	181.0495	181.0501	161.0390, 135.0489	C_9_H_9_O_4_	−3.3	Veratric acid^b^ (phenolic acid)	^25^
**2**	3.24	207.0295	207.0295	179.0376, 163.0399	C_10_H_7_O_5_	1.0	Fraxetin (coumarin)	^26^
**3**	3.63	593.1505	593.1506	473.1264, 353.0658	C_27_H_29_O_15_	−0.2	Vicenin 2^b^ (flavonoid)	^27^
**4**	3.77	311.0769	311.0767	193.0460, 178.0218, 149.0456, 134.0351	C_14_H_15_O_8_	0.6	Feruloyl threonic acid^b^ (phenolic acid)	^28^
**5**	3.82	179.0345	179.0344	135.0477	C_9_H_7_O_4_	0.6	Caffeic acid^b^,^a^ (phenolic acid)	^29^
**6**	4.04	351.1291	351.1291	249.0609	C_14_H_23_O_10_	0.0	Unknown	–
**7**	4.23	371.0977	371.0978	249.0576, 121.0303, 113.0255	C_16_H_19_O_10_	−0.3	Deacetylasperuloside^b^ (iridoid)	^30^
**8**	4.35	163.0391	163.0395	119.0493	C_9_H_7_O_3_	−2.5	p-coumaric acid^a^,^b^ (phenolic acid)	^31^
**9**	4.44	385.1144	385.1135	207.1059, 113.0237	C_17_H_21_O_10_	2.3	Sinapic acid-*O*-hexoside^b^	^32^
**10**	4.80	693.2028	693.2031	271.0932	C_32_H_37_O_17_	−0.4	Helonioside A (phenylpropanoid glycoside)	^33^
**11**	5.13	691.2618	691.2602	335.1241	C_34_H_43_O_15_	2.3	Unknown	–
**12**	5.41	271.0964	271.0970	–	C_16_H_15_O_4_	−2.2	Vestitol (flavonoid)	^34^
**13**	5.84	675.2654	675.2653	–	C_34_H_43_O_14_	0.1	Unknown	–
**14**	6.67	375.2758	375.2747	–	C_20_H_39_O_6_	−2.4	Fatty acid derivative^b^	–
**15**	6.90	373.2583	373.2590	–	C_20_H_37_O_6_	−1.9	Fatty acid derivative^b^	–
**16**	7.11	327.2161	327.2171	229.1406, 211.1334	C_18_H_31_O_5_	−3.1	Pinellic acid isomer^b^	^35^
**17**	7.34	371.2420	371.2434	–	C_20_H_35_O_6_	−3.8	Fatty acid derivative^b^	–
**18**	7.56	329.2317	329.2328	229.1441, 211.1323	C_18_H_33_O_5_	−3.3	Pinellic acid isomer^b^	^5^
**19**	7.94	359.2785	359.2797	–	C_20_H_39_O_5_	−3.3	Fatty acid derivative^b^	–

a Indicates comparison with standard.

b Indicates using fragments in MS/MS data.

### 3.2 Open field test

**Fig. 2:**
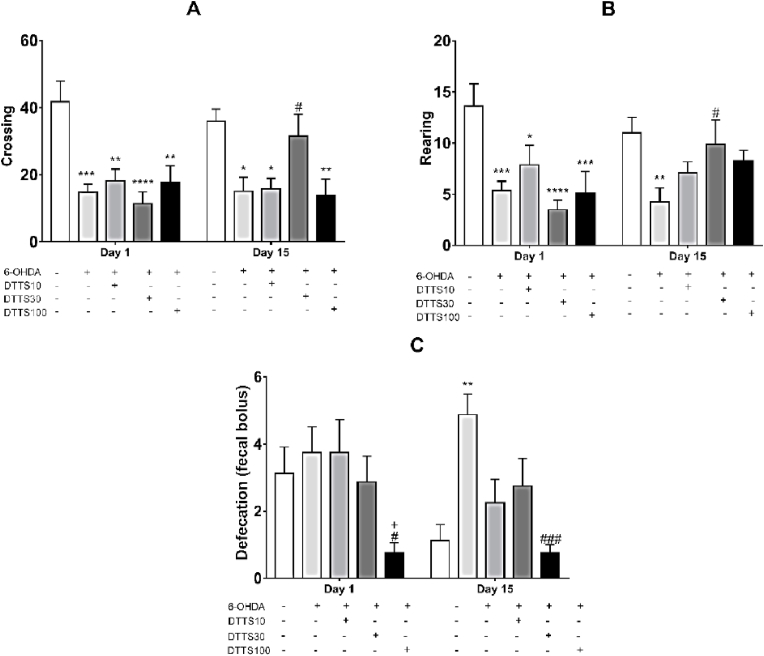
Results from behavioral analysis. In A, B and C, means of crossing, rearing and defecations, respectively, in the open field test at 1 and 15 days after the surgical microinjections of vehicle (Control) or 6-hydroxydopamine (6-OHDA) into dorsal striatum of rats (n = 8). In D, results of the cylinder test. Columns represent the mean e and bars indicate the SEM. DTTS: dry tea of *Tradescantia spathacea* at 10, 30 and 100 mg/kg in distillated water, p.o., during 30 days. * indicates significant difference from Control group (being * p < 0.05; **p < 0.01; ***p < 0.001 and ****p < 0.0001), # indicates significant difference from 6-OHDA (#p < 0.05 and ###p < 0.001), and + indicates significant difference from DTTS 10 (p < 0.05). Two-way analysis of variance with Bonferroni's post-tests.

At day 1 after surgery, all 6-OHDA groups presented a significant decrease in the mean of crossings when compared to the Control group, without significant differences between them. A similar result was observed after 15 days, except for the group DTTS 30, that presented mean values near to control, with a significant increase compared to DTTS 100 (Fig. 2A) (Interaction overall with values of F4,35 = 3.052 and p = 0.0294; treatment effect with F4,35 = 9.271 and p < 0.0001; and time effect with F1,35 = 0.5456 and p = 0.3724).

The groups 6-OHDA, DTTS 10, DTTS 30, and DTTS 100 showed a significant decrease in rearing compared to the Control group on the first day. On the other hand, after 15 days, the groups DTTS 10, DTTS 30 and DTTS 100, but not 6-OHDA, presented values similar to Control, and the group DTTS 30 showed a significant increase when compared to 6-OHDA (p = 0.0438, Fig. 2B) (Interaction overall with values of F4,35 = 4.11 and p = 0.0096; treatment effect with F4,35 = 4.191 and p = 0.0088; and time effect with values of F1,35 = 0.8547 and p = 0.3860). The DTTS 100 significantly counteracted this effect (Fig. 2C), presenting an anxiolytic effect. (Interaction overall with values of F4,35 = 1.897 and p = 0.1328; treatment effect with F4,35 = 6.479 and p = 0.0005; and time effect with F1,35 = 1.51 and p = 0.2273).

### 3.3 Cylinder test

**Fig. 3:**
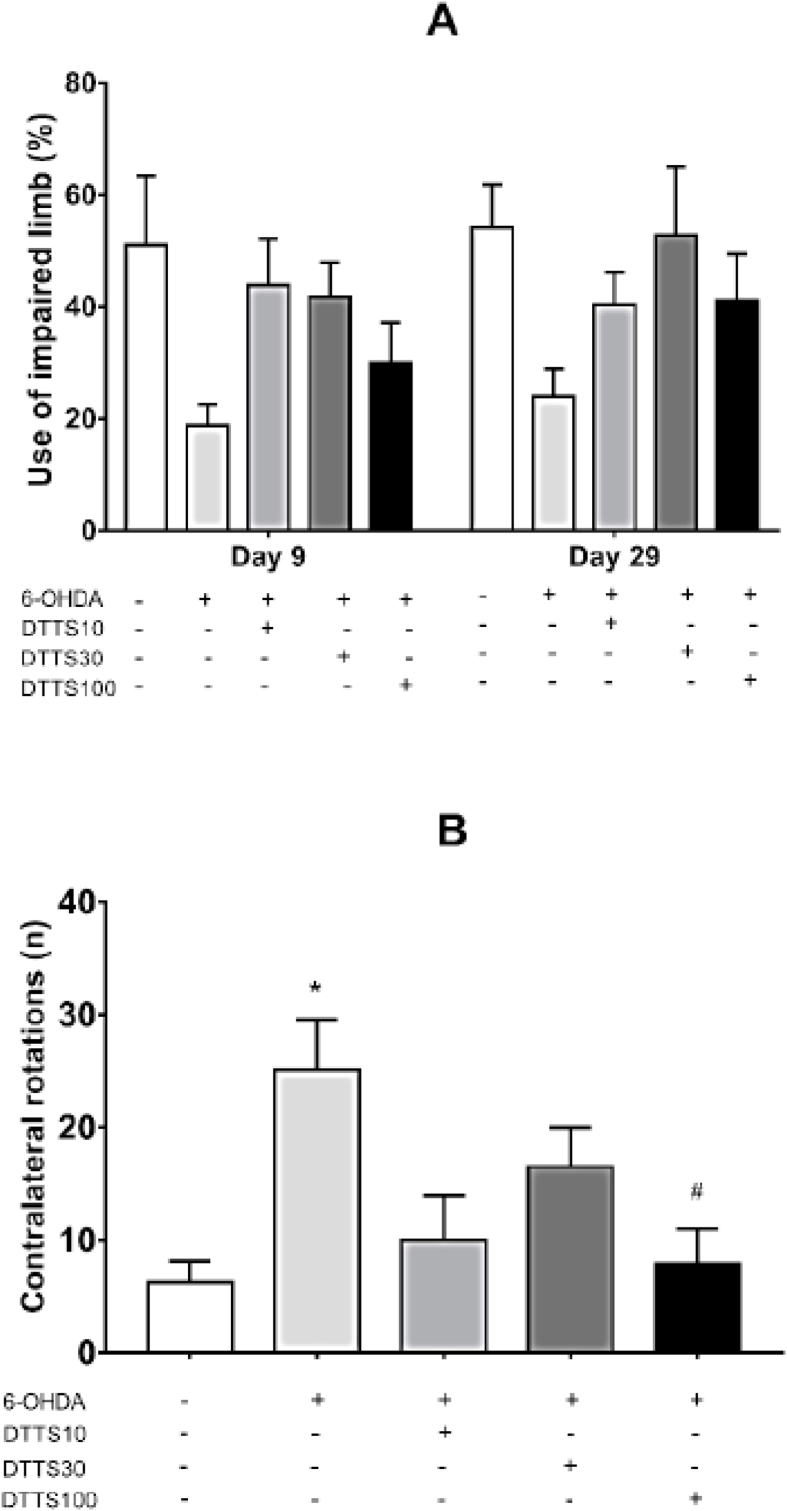
Results of behavioral analysis. In A, it measures the percentage of use of the contralateral limb, in the cylinder test at 9 and 29 days after surgical microinjections of vehicle (Control) or 6-hydroxydopamine (6-OHDA) in the dorsal striatum of rats (n = 8). Two-way analysis of variance with Bonferroni's post-tests. In B, it shows the 6-OHDA induced rotations towards the contralateral side, in the apomorphine-induced rotational behavior test, on the 30th day. Columns represent the mean and bars indicate the SEM. DTTS: *Tradescantia spathacea* dry tea at 10, 30 and 100 mg/kg in distilled water, v.o., for 30 days. * indicates a significant difference in relation to the Control group (with * p < 0.05), # indicates a significant difference in relation to 6-OHDA (#p < 0.05). Kruskal-Wallis with Dunn's post-test.

Contralateral forelimb analysis did not present statistical differences among groups on the 9th or 29th trial day. After 29 days, the 6-OHDA group remained with the lower mean values (less than 24% of correct alternations), similarly to the observed in the open field test (Fig. 3A). Only the Control and DTTS 30 groups presented such profile, but again, an anxiolytic effect of DTTS 100, with consequent reduction of spontaneous motor activity, cannot be discarded (Interaction overall with values of F4,35 = 0.286 and p = 0.8851; treatment effect with F4,35 = 4.443 and p = 0.0052; and time effect with F1,35 = 1.112 and p = 0.2990).

### 3.4 Apomorphine-induced rotational behavior

The 6-OHDA induced rotations towards the contralateral side (25.1 ± 4.4; Kruskal-Wallis test, p = 0.0117, Fig. 3B). This effect was lower in groups treated with DTTS 10 (10 ± 3.9; p = 0.1748), DTTS 30 (16.5 ± 3.5; p > 0.9999) and, in a significant manner, in the group DTTS 100 (7.8 ± 3.1; p = 0.0412). The means of the 6-OHDA group were significantly greater than those of the Control group (4.8 ± 1.4; p = 0.0302).

### 3.5 TH + cell counts and fiber density

**Fig. 4:**
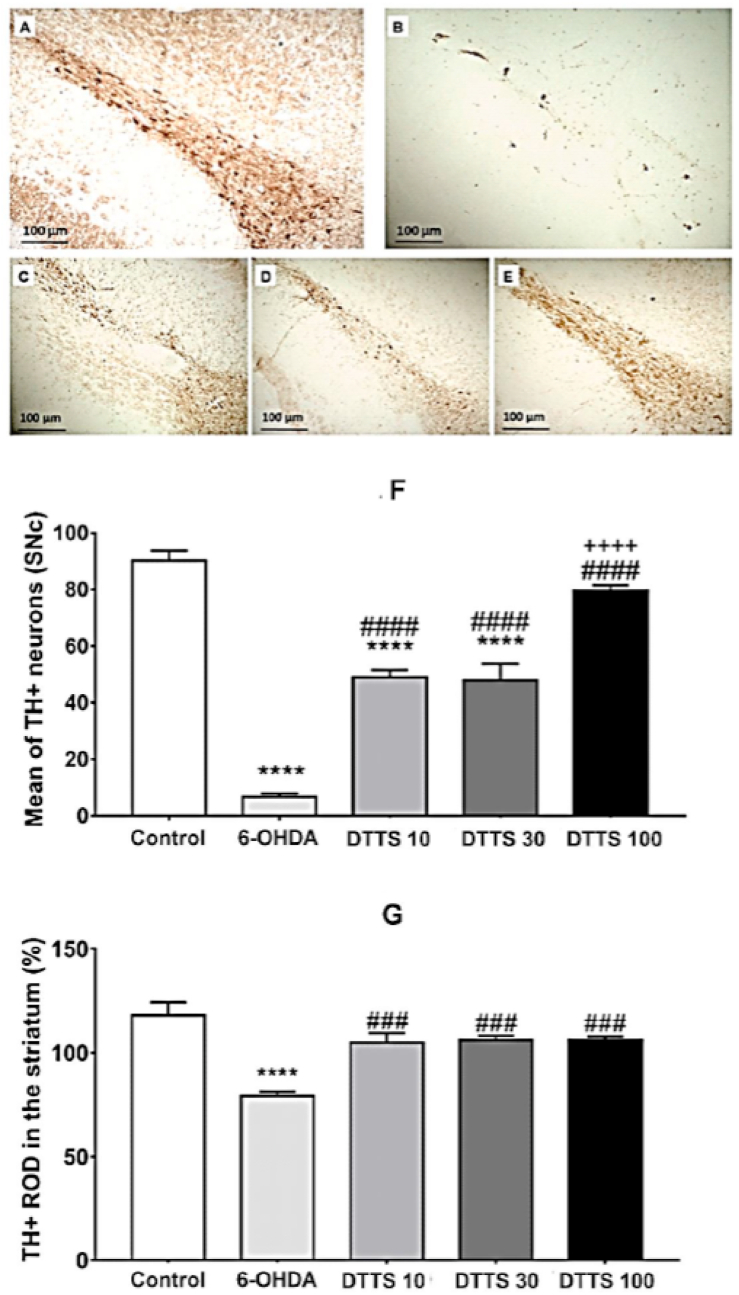
Photomicrographs of TH + neurons in the SNc of (A) Control group; (B) 6-OHDA group, (C) DTTS 10, (D) DTTS 30 and (E) DTTS 100 groups. F: quantitative results of the TH + neurons in the SNc as a percentage of the contralateral side, at 400× magnification. Photomicrographs of TH + relative optical density (ROD) in the striatum of (G) Control group; (H) 6-OHDA group, (I) DTTS 10, (J) DTTS 30 and (K) DTTS 100 groups. In L: quantitative results of the ROD in the striatum as a percentage of the contralateral side, at 400× magnification. Columns represent the mean e and bars indicate the SEM. Control: group of animals microinjected with vehicle and that received distillated water; 6-OHDA: group of animals microinjected with 6-hydroxydopamine and that received distillated water; DTTS 10, DTTS 30 and DTTS 100: group of animals microinjected with 6-hydroxydopamine and that received aqueous extract of *Tradescantia spathacea* at 10, 30 and 100 mg/kg, respectively. * indicates significant difference from Control group (****p < 0.0001), # indicates significant difference from 6-OHDA (###p < 0.001 and ####p < 0.0001) and + indicates difference from other doses of DTTS (++++ p < 0.0001). One-way analysis of variance with Bonferroni's post-tests.

In the SNc ipsilateral to the lesion site, it was observed a significant decrease in the mean number of TH + neurons in the groups 6-OHDA, DTTS 10, and DTTS 30 compared to Control, but not in the group DTTS 100 (Fig. 4A–E). Moreover, the neuroprotection was evident for all doses of DTTS, that promoted an increase in the mean of TH + neurons compared to 6-OHDA treated with the vehicle - the DTTS 100 showing an effect significantly greater than the other doses (F4,35 = 91.07, p < 0.0001, Fig. 4F).

Regarding the TH + fiber density in the striatum, it was observed a significant decrease in the 6-OHDA group from control. On the other hand, all DTTS groups showed statistical similarity to the Control group, at the same time that they presented a significant increase when compared to 6-OHDA (F4,25 = 13.95, p < 0.0001, Fig. 4G).

### 3.6 Glial (GFAP) response

**Fig. 5:**
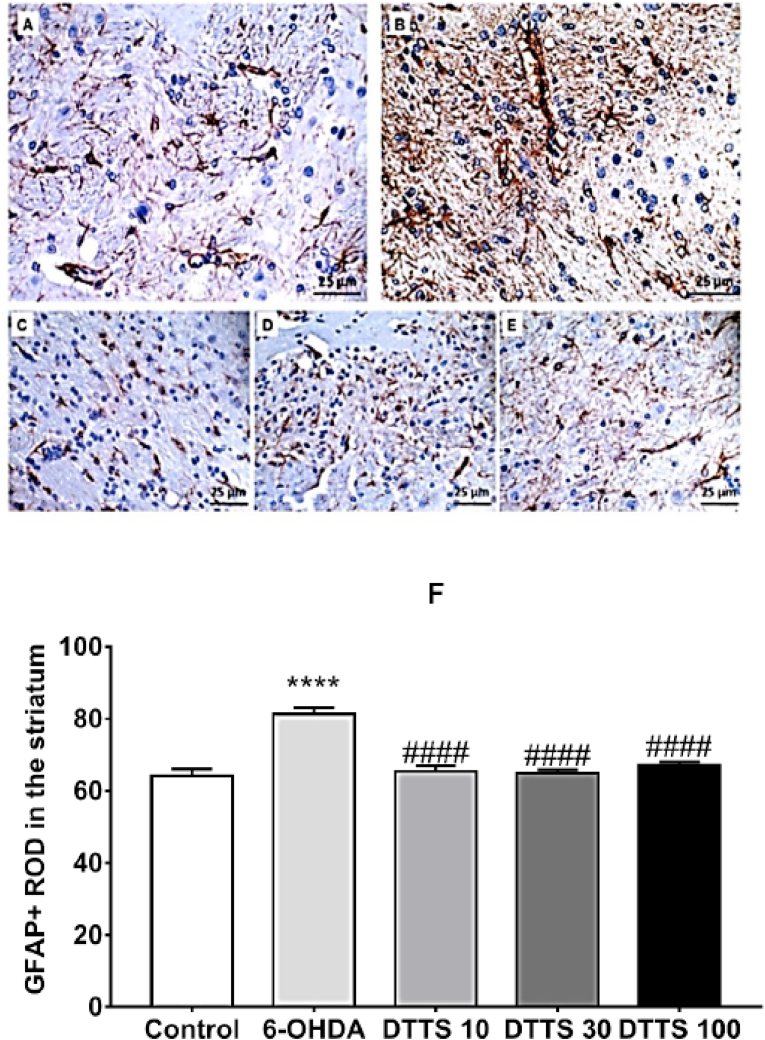
Photomicrographs of GFAP + cells in the striatum of (A) Control group; (B) 6-OHDA group, (C) DTTS 10, (D) DTTS 30 and (E) DTTS 100 groups. In F: quantitative results of the relative optical density (ROD) in the striatum as a percentage of the contralateral side, at 400× magnification. Columns represent the mean e and bars indicate the SEM. Control: group of animals microinjected with vehicle and that received distillated water; 6-OHDA: group of animals microinjected with 6-hydroxydopamine and that received distillated water; DTTS 10, DTTS 30 and DTTS 100: group of animals microinjected with 6-hydroxydopamine and that received aqueous extract of *Tradescantia spathacea* at 10, 30 and 100 mg/kg, respectively. **** indicates significant difference from Control group (p < 0.0001), and #### indicates significant difference from 6-OHDA (p < 0.0001).

The GFAP + cells were observed in the striatum (Fig. 5A–E) and it was found a significant increase of GFAP expression in the 6-OHDA group in comparison to the Control. The treatment with DTTS at 10, 30 and 100 mg/kg counteracted this effect since the mean values in these groups were significantly lower when compared to 6-OHDA (F4, 25 = 24.14, p < 0.0001, Fig. 5F).

## 4 Discussion

According to the literature, extracts of *T. spathacea* leaves can be obtained by distinct methods, such as decoction^36^ and by Soxhlet,^37^ however, the overall extraction yields (2.8, 8.4% and 3.4%, respectively) were reported to be lower than those found in our study. Moreover, DTTS values of phenolic compounds and flavonoids were higher than others previously reported: TPC of 2.3 mg/g,^7^ 8.5 mg/g,^38^ TFC of 0.7 mg/g,^7^ and 10.8 mg/g.^4^

Also, the *T. spathacea* showed relevant antioxidant activity (IC50), either by infusion (377.8 mg AA/100 g), decoction (309.0 mg AA/100 g), or methanolic extraction (486.4 mg AA/100 g),^22^ when compared to some species of Commelinaceae family, such as *Tradescantia pallida* (103 mg AA/100 g) and *Callisia fragrans* (262.5 mg AA/100 g).^4^ Taken together (elevated total phenolic and flavonoid contents and a low value of IC_50_), our results point to a high antioxidant chemical profile.

In DTTS, phenolic acids,^25^ coumarin,^26^ flavonoids,^34^ iridoid,^30^ phenylpropanoid glycoside^33^ and fatty acid derivative,^35^ were identified and reported to have neuroprotective activities. In the literature, only p-coumaric acid had already been identified in *T. spathacea*.^5^ Then the other 15 biocompounds were tentatively identified in *T. spathacea* for the first time in this study.

In the biological assay, 6-OHDA induces motor deficits in a manner closely related to the level of dopaminergic cell loss and dopamine depletion,^39^ and it was observed in 24 h (initial functional symptoms)^40^ and 15 days after surgery (more stabilized lesion signs).^41^ These effects were counteracted over time by DTTS at 30 mg/kg, indicating a neuroprotective effect. This can be attributed to the synergistic effect between the bioactive compounds present in *T. spathacea*; however, a dose of DTTS 10 mg/kg was not enough for the animals to show significant recovery of these movements. At the highest dose (DTTS100), there was also no increase in spontaneous exploratory behavior, yet it cannot be concluded that there was no neuroprotection, as immunohistochemical analyzes indicate preservation of striatal fibers and dopaminergic neurons, in addition to a reduction in astrocytic occurrence. Therefore, it is suggested that animals treated daily with a dose of 100 mg/kg presented lethargy due to the presence of bioactive compounds in *T. spathacea* which are associated with the anxiolytic effect.

Moreover, 6-OHDA induced an increase in the mean of the fecal bolus after 15 days from surgery, an indicator of anxiety behavior.^42^ Besides, *T. spathacea* is one of the medicinal plants used to treat anxiety in Mexico.^43^

Among these compounds that may have influenced the reduction of spontaneous exploratory behavior in the open field test, vicenin-2 and fraxetine can be mentioned. Vicenin-2 is one of the flavonoids present in DTTS (compound 3), and the main ingredient of Passiflora edulis, which has been related to antidepressant action, acting in synergy with other compounds.^44^ In addition to fraxetin, a coumarin is also present in DTTS (compound 2), which was associated with the attenuation of anxious and depressive behavior *in vivo*, in addition to acting in an anti-inflammatory mechanism by mediating expression of interleukins (IL-1β, IL-6), TNF-α, nitric oxide and microglia.^45^^,^^46^

For cylinder test, they are considered healthy the animals with at least 50% of correct contralateral alternations,^47^ and only the Control and DTTS 30 groups presented such profile, but again, an anxiolytic effect of DTTS 100, with consequent reduction of spontaneous motor activity, cannot be discarded.

If spontaneous activity assessed by open field and cylinder tests can be affected by emotional aspects, the apomorphine-induced rotational behavior, on the other hand, is elicited only when dopamine depletion is up to 80%,^10^ as a result of a dopamine receptor hypersensitivity after lesion.^48^

It is expected a loss of about 52% of dopaminergic neurons and a consequent loss of striatal dopaminergic fiber density, 4 weeks after 6-OHDA striatal injections.^49^ In the present study, this effect was observed in the 6-OHDA group and was counteracted by DTTS in both in SNc and striatum, the effect being more evident (but not exclusive) for DTTS 100. In fact, DTTS promoted an increase in the percentage of surviving TH + cell bodies and axons. Thus, a slight sedative effect maybe considers for DTTS 100, since it was not able to increase crossings after lesion, despite its neuroprotective effect seemed in the histological evaluation.

Although the role of reactive astrocytes in PD is still unclear, since they can prevent or activate neurodegeneration,^50^ the astroglial reaction until 3–4 weeks after 6-OHDA injection has been well documented^51^ and accompanies the progressive loss of dopaminergic neurons.^20^ In physiological conditions, the astrocytes release neurotrophic factors and are critical for the maintaining of the extracellular environment and for conserving the basal levels of glutamate and gamma-aminobutyric acid (GABA).^52^ After dopaminergic lesion, reactive astrocytes show an up-regulation of GFAP (an intermediate filament protein up-regulated by the astrocyte hypertrophy), glutamine synthetase (which protects neurons by metabolizing the astrocytic glutamate into glutamine), S100β (a calcium-binding protein), NDRG2 (a tumor suppressor protein associated to astrocyte proliferation) and vimentin (intermediate filament protein present in migrating astrocytes and in glial scars).^53^

Although the role of autophagy in astrocytes has not been fully elucidated in Parkinson's disease, it is known that adequate modulation of astrocytic autophagy may be associated with a protective mechanism,^54^^,^^55^ attenuating protein aggregates, such as α-synuclein,^56^^,^^57^ reducing reactive oxygen species and consequently oxidative stress and neuroinflammation.^58^ Some polyphenols are being associated with properties that modulate autophagy and neuroprotection, therefore the neuroprotective effect of DTTS was evident and it may be related to its chemical composition. Among these compounds, caffeic acid (compound 5) can be mentioned, which reduced A53T α-synuclein, activating JNK/Bcl-2-mediated autophagy in an *in vitro* model, and protected dopaminergic neurons in an *in vivo* model of PD.^59^ Suggesting that the mediation of autophagy through bioactive compounds may have been one of the mechanisms of this experimental model.

For example, fraxetin (compound 2) presented antioxidation and anti-apoptotic properties in human neuroblastoma cells exposed to rotenone by increasing endogenous levels of reduced glutathione and decreasing activated caspase 3 and 9, and the formation of intracellular oxygen species.^60^ In an animal model of rotenone-induced PD, treatment with caffeic acid (compound 5) improved TH immunostaining, attenuated motor deficits, and reduced the expression of microglial cells and the production of inflammatory mediators such as COX-2, iNOS, and NFκB.^61^

The veratric acid (compound 1) is a phenolic acid with reported anti-inflammatory and antioxidant activities and can inhibit LPS-induced production of pro-inflammatory cytokines such as IL-6, IL-8, TNF-α, and IL-1β, as well as iNOS and COX-2 expression.^62^ Veratric acid derivatives were tyrosinase inhibitors and free radical scavengers.^63^

The p-coumaric acid (compound 8) presented neuroprotective effects in animal models of ischemia, with strong anti-oxidant and anti-apoptotic features.^64^ The polyphenols detected in the *Lycium ruthenicum,* including caffeic acid (compound 5) and p-coumaric acid (compound 8), also present in DTTS, protected neuronal cells (PC-12) from cytotoxicity, preventing apoptosis by oxidative stress.^65^

Flavonoids, which are considered therapeutic agents for neurodegenerative diseases^66^ including PD, present the ability to modulate cellular signaling pathways, reduce NADPH, decrease the production of intracellular oxygen species and inhibit the release of cytokines.^67^ Vestitol (compound 12), for example, presents well documented anti-inflammatory effects and diminished the activation of NF-κB and Erk 1/2 pathways, and induced macrophages into M2-like polarization.^68^ This compound was highly presented in a red propolis extract with a neuroprotective effect against nerve injury.^69^

The fatty acid derivatives reduced neurodegeneration, increased tyrosine hydroxylase levels, and prevented α-synuclein aggregation, in addition to attenuating behavioral damage induced by 1-methyl-4-phenyl-1,2,3,6-tetrahydropyridine (MPTP) in mice.^70^ Pinellic acid (isomer compounds 16 and 18), a derived fatty acid, inhibited prostaglandin D₂ and nitric oxide production *in vitro*.^71^

Iridoids present anti-inflammatory and neuroprotective effects. For instance, loganine reduced oxidative stress and apoptosis induced by 1‐methyl‐4‐fenillpyridinium (MPP), regulated tyrosine hydroxylase, and expressions of neurotrophic signals including IGF‐1R, GLP‐1R, p‐Akt, BDNF.^72^

## 5 Conclusions

In this research, we found 15 biocompounds that had not been previously reported in *T. spathacea*. Based on the results, we show in an unprecedented way that these dry tea biocompounds from *T. spathacea* leaves allowed a neuroprotective effect in the neurodegenerative loss of the nigrostriatal dopaminergic pathway. The dry tea of *T. spathacea* leaves reverted the motor symptoms and all doses protected TH + cells and reduced astroglial response. The DTTS is, therefore, a potential therapeutic approach to be considered for future studies of Parkinson's Disease.

## Declaration of competing interest

The authors declare that they have no known competing financial interests or personal relationships that could have appeared to influence the work reported in this paper.

## References

[1] Sharma R., Padwad Y. (2020). Perspectives of the potential implications of polyphenols in influencing the interrelationship between oxi-inflammatory stress, cellular senescence and immunosenescence during aging. Trends Food Sci Technol.

[2] Simsek M., Süfer Ö. (2021). Infusion of walnut (Juglans regia L.) shell tea: multi response optimization and antioxidant potential. J Appl Res Med Aromat Plants.

[3] Kyaw E.H., Kato-Noguchi H. (2021). Assessment of allelopathic activity of Tradescantia spathacea Sw. for weed control. Biol Futura.

[4] Tan J.B.L., Lim Y.Y., Lee S.M. (2014). Rhoeo spathacea (Swartz) Stearn leaves, a potential natural food colorant. J Funct Foods.

[5] García-Varela R., Fajardo Ramírez O.R., Serna-Saldivar S.O., Altamirano J., Cardineau G.A. (2016). Cancer cell specific cytotoxic effect of Rhoeo discolor extracts and solvent fractions. J Ethnopharmacol.

[6] González-Avila M., Arriaga-Alba M., de la Garza M. (2003). Antigenotoxic, antimutagenic and ROS scavenging activities of a Rhoeo discolor ethanolic crude extract. Toxicol Vitro.

[7] Sánchez-Roque Y., Ayora-Talavera G., Rincón R. (2017). The flavonoid fraction from Rhoeo discolor leaves acts antiviral against influenza A virus. Record Nat Prod.

[8] Hosamani R., Krishna G., Muralidhara (2016). Standardized Bacopa monnieri extract ameliorates acute paraquat-induced oxidative stress, and neurotoxicity in prepubertal mice brain. Nutr Neurosci.

[9] Bridi J.C., Hirth F. (2018). Mechanisms of α-synuclein induced synaptopathy in Parkinson's disease. Front Neurosci.

[10] Lima F.A.V., Joventino I.P., Joventino F.P. (2017). Neuroprotective activities of spirulina platensis in the 6-OHDA model of Parkinson's disease are related to its anti-inflammatory effects. Neurochem Res.

[11] Takahashi S., Mashima K. (2022). Neuroprotection and disease modification by astrocytes and microglia in Parkinson disease. Antioxidants.

[12] Cano A., Fonseca E., Ettcheto M. (2021). Epilepsy in neurodegenerative diseases: related drugs and molecular pathways. Pharmaceuticals.

[13] Hussein R.M., Mohamed W.R., Omar H.A. (2018). A neuroprotective role of kaempferol against chlorpyrifos-induced oxidative stress and memory deficits in rats via GSK3β-Nrf2 signaling pathway. Pestic Biochem Physiol.

[14] Li X., Wang H., Wen G. (2018). Neuroprotection by quercetin via mitochondrial function adaptation in traumatic brain injury: PGC-1α pathway as a potential mechanism. J Cell Mol Med.

[15] Almutairi M.M., Alanazi W.A., Alshammari M.A. (2017). Neuro-protective effect of rutin against Cisplatin-induced neurotoxic rat model. BMC Compl Alternative Med.

[16] Singh N.A., Mandal A.K., Khan Z.A. (2016). Potential neuroprotective properties of epigallocatechin-3-gallate (EGCG). Nutr J.

[17] Wang Y.L., Ju B., Zhang Y.Z. (2017). Protective effect of curcumin against oxidative stress-induced injury in rats with Parkinson's disease through the Wnt/β-catenin signaling pathway. Cell Physiol Biochem.

[18] Magalingam K.B., Radhakrishnan A., Haleagrahara N. (2016). Protective effects of quercetin glycosides, rutin, and isoquercitrin against 6-hydroxydopamine (6-OHDA)-induced neurotoxicity in rat pheochromocytoma (PC-12) cells. Int J Immunopathol Pharmacol.

[19] Datta I., Ganapathy K., Razdan R., Bhonde R. (2018). Location and number of astrocytes determine dopaminergic neuron survival and function under 6-OHDA stress mediated through differential BDNF release. Mol Neurobiol.

[20] Mori M.A., Delattre A.M., Carabelli B. (2018). Neuroprotective effect of omega-3 polyunsaturated fatty acids in the 6-OHDA model of Parkinson's disease is mediated by a reduction of inducible nitric oxide synthase. Nutr Neurosci.

[21] Parkinson's Disease Cacabelos R. (2017). From pathogenesis to pharmacogenomics. Int J Mol Sci.

[22] Tan J.B., Lim Y.Y., Lee S.M. (2015). Antioxidant and antibacterial activity of Rhoeo spathacea (Swartz) Stearn leaves. J Food Sci Technol.

[23] Sampaio T.B., Pinton S., da Rocha J.T., Gai B.M., Nogueira C.W. (2017). Involvement of BDNF/TrkB signaling in the effect of diphenyl diselenide on motor function in a Parkinson's disease rat model. Eur J Pharmacol.

[24] Padovan-Neto F.E., Cavalcanti-Kiwiatkoviski R., Carolino R.O., Anselmo-Franci J., Del Bel E. (2015). Effects of prolonged neuronal nitric oxide synthase inhibition on the development and expression of L-DOPA-induced dyskinesia in 6-OHDA-lesioned rats. Neuropharmacology.

[25] Jia Q., Zhang S., Zhang H. (2020). A comparative study on polyphenolic composition of berries from the Tibetan plateau by UPLC-Q-orbitrap MS system. Chem Biodivers.

[26] Saleem H., Zengin G., Locatelli M. (2019). In vitro biological propensities and chemical profiling of Euphorbia milii Des Moul (Euphorbiaceae): a novel source for bioactive agents. Ind Crop Prod.

[27] Farooq M.U., Mumtaz M.W., Mukhtar H. (2020). UHPLC-QTOF-MS/MS based phytochemical characterization and anti-hyperglycemic prospective of hydro-ethanolic leaf extract of Butea monosperma. Sci Rep.

[28] Wang T.M., Fu Y., Yu W.J. (2017). Identification of polar constituents in the decoction of Juglans mandshurica and in the medicated egg prepared with the decoction by HPLC-Q-TOF MS^2^. Molecules.

[29] Campos D.A., Ribeiro T.B., Teixeira J.A., Pastrana L., Pintado M.M. (2020). Integral valorization of pineapple (*Ananas comosus* L.) by-products through a green chemistry approach towards added value ingredients. Foods.

[30] Amessis-Ouchemoukh N., Abu-Reidah I.M., Quirantes-Piné R. (2014). Tentative characterisation of iridoids, phenylethanoid glycosides and flavonoid derivatives from Globularia alypum L. (Globulariaceae) leaves by LC-ESI-QTOF-MS. Phytochem Anal.

[31] Amaya-Cruz D.M., Pérez-Ramírez I.F., Delgado-García J., Mondragón-Jacobo C., Dector-Espinoza A., Reynoso-Camacho R. (2019). An integral profile of bioactive compounds and functional properties of prickly pear (Opuntia ficus indica L.) peel with different tonalities. Food Chem.

[32] Lorenzo J.M., Mousavi Khaneghah A., Gavahian M. (2019). Understanding the potential benefits of thyme and its derived products for food industry and consumer health: from extraction of value-added compounds to the evaluation of bioaccessibility, bioavailability, anti-inflammatory, and antimicrobial activities. Crit Rev Food Sci Nutr.

[33] Zou W., Zhou H., Hu J. (2017). Rhizoma Smilacis Glabrae inhibits pathogen-induced upper genital tract inflammation in rats through suppression of NF-κB pathway. J Ethnopharmacol.

[34] Yerlikaya S., Baloglu M.C., Diuzheva A., Jekő J., Cziáky Z., Zengin G. (2019). Investigation of chemical profile, biological properties of Lotus corniculatus L. extracts and their apoptotic-autophagic effects on breast cancer cells. J Pharmaceut Biomed Anal.

[35] Barragán-Zarate G.S., Lagunez-Rivera L., Solano R. (2020). Prosthechea karwinskii, an orchid used as traditional medicine, exerts anti-inflammatory activity and inhibits ROS. J Ethnopharmacol.

[36] Rosales-Reyes T., de la Garza M., Arias-Castro C. (2008). Aqueous crude extract of Rhoeo discolor, a Mexican medicinal plant, decreases the formation of liver preneoplastic foci in rats. J Ethnopharmacol.

[37] Mena-Rejon G., Caamal-Fuentes E., Cantillo-Ciau Z., Cedillo-Rivera R., Flores-Guido J., Moo-Puc R. (2009). In vitro cytotoxic activity of nine plants used in Mayan traditional medicine. J Ethnopharmacol.

[38] García-Varela R., García-García R.M., Barba-Dávila B.A., Fajardo-Ramírez O.R., Serna-Saldívar S.O., Cardineau G.A. (2015). Antimicrobial activity of Rhoeo discolor phenolic rich extracts determined by flow cytometry. Molecules.

[39] Carvalho M.M., Campos F.L., Coimbra B. (2013). Behavioral characterization of the 6-hydroxidopamine model of Parkinson's disease and pharmacological rescuing of non-motor deficits. Mol Neurodegener.

[40] Martins W.B., Rodrigues S.A., Silva H.K. (2016). Neuroprotective effect of Portulaca oleracea extracts against 6-hydroxydopamine-induced lesion of dopaminergic neurons. An Acad Bras Cienc.

[41] Antipova V.A., Holzmann C., Schmitt O., Wree A., Hawlitschka A. (2017). Botulinum neurotoxin A injected ipsilaterally or contralaterally into the striatum in the rat 6-OHDA model of unilateral Parkinson's disease differently affects behavior. Front Behav Neurosci.

[42] Su R.J., Zhen J.L., Wang W., Zhang J.L., Zheng Y., Wang X.M. (2018). Time-course behavioral features are correlated with Parkinson's disease-associated pathology in a 6-hydroxydopamine hemiparkinsonian rat model. Mol Med Rep.

[43] Gutiérrez S.L.G., Chilpa R.R., Jaime H.B. (2014). Medicinal plants for the treatment of “nervios”, anxiety, and depression in Mexican Traditional Medicine. Rev Bras Farmacogn.

[44] Retamozo M.H., Silva C.C., Tamayose C.I. (2023). Chemical constituents from leaves of *Baccharis sphenophylla* (Asteraceae) and their antioxidant effects. Plants.

[45] Deng S.J., Ge J.W., Xia S.N. (2022). Fraxetin alleviates microglia-mediated neuroinflammation after ischemic stroke. Ann Transl Med.

[46] Ahmed Z., Tokhi A., Arif M. (2023). Fraxetin attenuates disrupted behavioral and central neurochemical activity in a model of chronic unpredictable stress. Front Pharmacol.

[47] Boix J., Padel T., Paul G. (2015). A partial lesion model of Parkinson's disease in mice--characterization of a 6-OHDA-induced medial forebrain bundle lesion. Behav Brain Res.

[48] Konieczny J., Czarnecka A., Lenda T., Kamińska K., Antkiewicz-Michaluk L. (2017). The significance of rotational behavior and sensitivity of striatal dopamine receptors in hemiparkinsonian rats: a comparative study of lactacystin and 6-OHDA. Neuroscience.

[49] Silva T.P., Poli A., Hara D.B., Takahashi R.N. (2016). Time course study of microglial and behavioral alterations induced by 6-hydroxydopamine in rats. Neurosci Lett.

[50] Heimann G., Sirko S. (2019). Investigating Age-related changes in proliferation and the cell division repertoire of parenchymal reactive astrocytes. Methods Mol Biol.

[51] Haas S.J., Zhou X., Machado V., Wree A., Krieglstein K., Spittau B. (2016). Expression of Tgfβ1 and inflammatory markers in the 6-hydroxydopamine mouse model of Parkinson's disease. Front Mol Neurosci.

[52] Yelkenli İ.H., Ulupinar E., Korkmaz O.T. (2016). Modulation of corpus striatal neurochemistry by astrocytes and vasoactive intestinal peptide (VIP) in Parkinsonian rats. J Mol Neurosci.

[53] Morales I., Sanchez A., Rodriguez-Sabate C., Rodriguez M. (2016). The astrocytic response to the dopaminergic denervation of the striatum. J Neurochem.

[54] Bhushan B., Singh N.K. (2023).

[55] Janda E., Lascala A., Carresi C. (2015). Parkinsonian toxin-induced oxidative stress inhibits basal autophagy in astrocytes via NQO2/quinone oxidoreductase 2: implications for neuroprotection. Autophagy.

[56] Lv Q.-K., Tao K.-X., Wang X.-B. (2023). Role of α-synuclein in microglia: autophagy and phagocytosis balance neuroinflammation in Parkinson's disease. Inflamm Res.

[57] Rocha S.M., Kirkley K.S., Chatterjee D., Aboellail T.A., Smeyne R.J., Tjalkens R.B. (2023). Microglia-specific knock-out of NF-κB/IKK2 increases the accumulation of misfolded α-synuclein through the inhibition of p62/sequestosome-1-dependent autophagy in the rotenone model of Parkinson's disease. Glia.

[58] Janda E., Parafati M., Martino C. (2023). Autophagy and neuroprotection in astrocytes exposed to 6-hydroxydopamine is negatively regulated by NQO2: relevance to Parkinson's disease. Sci Rep.

[59] Zhang Y., Wu Q., Zhang L. (2019). Caffeic acid reduces A53T α-synuclein by activating JNK/Bcl-2-mediated autophagy in vitro and improves behaviour and protects dopaminergic neurons in a mouse model of Parkinson's disease. Pharmacol Res.

[60] Sánchez-Reus M.I., Peinado, Molina-Jiménez M.F., Benedí J. (2005). Fraxetin prevents rotenone-induced apoptosis by induction of endogenous glutathione in human neuroblastoma cells. Neurosci Res.

[61] Zaitone S.A., Ahmed E., Elsherbiny N.M. (2019). Caffeic acid improves locomotor activity and lessens inflammatory burden in a mouse model of rotenone-induced nigral neurodegeneration: relevance to Parkinson's disease therapy. Pharmacol Rep.

[62] Wang Q.B., Sun L.Y., Gong Z.D., Du Y. (2016). Veratric acid inhibits LPS-induced IL-6 and IL-8 production in human gingival fibroblasts. Inflammation.

[63] Dehghani Z., Khoshneviszadeh M., Khoshneviszadeh M., Ranjbar S. (2019). Veratric acid derivatives containing benzylidene-hydrazine moieties as promising tyrosinase inhibitors and free radical scavengers. Bioorg Med Chem.

[64] Sakamula R., Thong-Asa W. (2018). Neuroprotective effect of p-coumaric acid in mice with cerebral ischemia reperfusion injuries. Metab Brain Dis.

[65] Gao H., Yuan X., Wang Z., Gao Q., Yang J. (2020). Profiles and neuroprotective effects of Lycium ruthenicum polyphenols against oxidative stress-induced cytotoxicity in PC12 cells. J Food Biochem.

[66] Cano A., Ettcheto M., Chang J.H. (2019). Dual-drug loaded nanoparticles of Epigallocatechin-3-gallate (EGCG)/Ascorbic acid enhance therapeutic efficacy of EGCG in a APPswe/PS1dE9 Alzheimer's disease mice model. J Contr Release.

[67] Solanki I., Parihar P., Parihar M.S. (2016). Neurodegenerative diseases: from available treatments to prospective herbal therapy. Neurochem Int.

[68] Bueno-Silva B., Rosalen P.L., Alencar S.M., Mayer M.P.A. (2020). Vestitol drives LPS-activated macrophages into M2 phenotype through modulation of NF-κB pathway. Int Immunopharm.

[69] Barbosa R.A., Nunes T.L., Nunes T.L. (2016). Hydroalcoholic extract of red propolis promotes functional recovery and axon repair after sciatic nerve injury in rats. Pharm Biol.

[70] Cordaro M., Siracusa R., Crupi R. (2018). 2-Pentadecyl-2-Oxazoline reduces neuroinflammatory environment in the MPTP model of Parkinson disease. Mol Neurobiol.

[71] Choi H.G., Park Y.M., Lu Y., Chang H.W., Na M., Lee S.H. (2013). Inhibition of prostaglandin D₂ production by trihydroxy fatty acids isolated from Ulmus davidiana var. japonica. Phytother Res.

[72] Tseng Y.T., Lin W.J., Chang W.H., Lo Y.C. (2019). The novel protective effects of loganin against 1-methyl-4-phenylpyridinium-induced neurotoxicity: enhancement of neurotrophic signaling, activation of IGF-1R/GLP-1R, and inhibition of RhoA/ROCK pathway. Phytother Res.

